# Electrical Conductivity and pH Are Two of the Main Factors Influencing the Composition of Arbuscular Mycorrhizal Fungal Communities in the Vegetation Succession Series of Songnen Saline-Alkali Grassland

**DOI:** 10.3390/jof9090870

**Published:** 2023-08-23

**Authors:** Lin-Lin Fang, Ya-Jie Liu, Zi-He Wang, Xiao-Yu Lu, Jin-Hua Li, Chun-Xue Yang

**Affiliations:** College of Landscape Architecture, Northeast Forestry University, Harbin 150040, China; fangll1223@163.com (L.-L.F.); 16653316925@163.com (Y.-J.L.); ziwanghe1008@163.com (Z.-H.W.); luxiaoyu0602@163.com (X.-Y.L.); tsinghuali0508@163.com (J.-H.L.)

**Keywords:** AMF, community succession, ecological matching phenomenon, Songnen saline-alkali grassland

## Abstract

Arbuscular mycorrhizal fungi (AMF) are widely distributed microorganisms in the soil, playing an important role in vegetation succession, plant community diversity, and improving soil physicochemical properties. In this study, morphological identification and high-throughput sequencing technology were used to comprehensively analyze the AMF community composition and diversity at different succession stages of Songnen saline-alkali grassland. To determine the root colonization status of plants collected in the field, a colonization system was established using late-succession plants as host plants to verify the existence of mycorrhizal symbiosis and the matching phenomenon of AMF in Songnen saline-alkali grassland. The results indicated that both morphological methods and high-throughput sequencing technology showed that glomus was the dominant genus of AMF in Songnen saline grassland. Redundancy analysis (RDA) and linear regression analysis showed that electrical conductivity (EC) and pH were the main environmental factors affecting AMF species diversity and community structure in the succession sequence of Songnen saline grassland. In addition, the results of root colonization identification and the colonization system test in the field showed that AMF successfully colonized vegetation at different succession stages and had mycorrhizal symbiosis. The results of this study could help to understand the AMF community of Songnen saline-alkali grassland as well as provide a reference and basis for optimizing the AMF community structure of Songnen saline-alkali grassland through human intervention in the future and using mycorrhizal technology to restore and rebuild the degraded ecosystem of Songnen saline-alkali grassland.

## 1. Introduction

Songnen saline-alkali grassland is located on the Songnen Plain, one of the three major plains in northeast China, with a total area of 3.42 × 10^6^ square kilometers, accounting for approximately 19.4% of the whole region and expanding at a rate of 1.4% per year [1,2,3]. The saline-alkali soil was mainly distributed in the semi-arid to arid areas in the west of the plain [4]. From the perspective of soil salinization characteristics, the salinization soil composition of saline soil in Songnen saline grassland was mainly NaHCO_3_ and Na_2_CO_3_, and the soil salinization process had the characteristics of simultaneous occurrence of salinization and sodium (alkalinization), and simultaneous deterioration of soil chemical and physical properties [5,6]. On the Songnen saline-alkali grassland, with the degree of soil salinization increasing from high to low, the dominant population was *Suaeda glauca*, *Puccinellia tenuiflora*, and *Leymus chinensis*, which constituted the community succession series of Songnen saline-alkali grassland [7]. At the same time, we often see concentric series of space succession consisting of light spots to communities of *S. glauca*, *P. tenuiflora*, and *L. chinensis*, which is actually a microcosm of the time succession process in the same place [8].

The degree of soil salinization is an important factor affecting plant growth, and high concentrations of salt or alkali will cause plant yield reduction or death [9,10]. Plant salinity and alkali damage are not only obstacles to agriculture and economic development in the saline-alkali grassland of Songnen but also a worldwide problem [11]. In order to better solve this problem, different physical, chemical, and biological methods have been adopted, and studies have found that the symbiosis of plants and AMF can effectively improve plant salinity tolerance [12]. As one of the important microorganisms in the soil, AMF can form a symbiotic relationship with more than 80% of higher plants [13]. From the perspective of biological evolution, the symbiosis of arbuscular mycorrhizal fungi and plants in saline-alkali land is conducive to the survival of both sides in a saline-alkali environment and plays an important role in promoting plant growth and improving plant salinity tolerance. AMF in saline-alkali soils were abundant and have been demonstrated [14,15] to play an important role in plant community establishment, community diversity, and the acceleration of ecosystem plant succession by improving soil physicochemical properties [16,17].

In the current research, the diversity of AMF in the soil is often analyzed by spore morphological methods, and classical morphological identification has become the basis for the application of single species [18]. However, there may be differences between the root soil and the community in the plant root system, and the composition of the microflora in the roots of the plant directly reflects the symbiosis between plants and AMF, which indicates that the morphological method has certain limitations in mycorrhizal research. The rapid development of high-throughput sequencing technology provides technical support for the accurate and efficient study of microbiota structure, and at the same time, it also creates the possibility of analysis of AMF communities in plant roots [19]. Based on this, the combination of the spore morphology identification method and high-energy sequencing technology in this study helps us to understand the diversity of AMF communities in Songnen saline grassland.

Recently, the analysis of previous studies on mycorrhizal function found that the mycorrhizal effect of mixed cultures was better than that of single species [20], and the host biomass increased greatly when plants, AMF, and soil were combined in the same domain, suggesting that there was local adaptation or ecological matching in the process of AMF-plant symbiosis [21,22]. Plants in different succession periods responded differently to AMF, and late plants were more dependent on AMF than early plants [23]. Previous studies had shown that AMF networks can mediate material transport between plants and enhance or weaken competition or reciprocity between plants [24,25]. *L. chinensis* was often grown as a mixture of late-successional plants with associated species such as *Chloris virgata*, *Medicago sativa*, and *Lepidium apetalum*, and the mycorrhizal network system had been shown to work between host plants and associated species [26,27]. However, there is still a lack of research on the local adaptability of AMF resources in saline-alkali land. Clarifying the AMF-plant symbiosis adaptability in different succession stages of Songnen saline-alkali grassland is crucial for the application of mycorrhizal technology to the ecological restoration of saline-alkali land.

Both plants in the saline grassland of Songnen and AMF exist and function in a community manner. So, are there differences in the community structure and diversity of AMF at different succession stages? What factors drive the succession of AMF communities? Is there a local adaptation or ecological matching phenomenon in AMF-plant symbiosis? In order to better solve these problems, we used high-throughput sequencing technology supplemented by morphological identification to analyze the structure characteristics and diversity of AMF communities in the root enclosure and plant roots at different succession stages in the Songnen saline-alkali grassland. The relationship between AMF community structure and soil physicochemical properties at different succession stages in Songnen saline-alkali grassland was clarified, and on this basis, *L. chinensis*, a plant in the late succession stage of Songnen saline-alkali grassland, was used as the host plant to further clarify whether there is an ecological matching effect of AMF communities in different vegetation community succession stages, which provides a more reliable scientific basis for optimizing the AMF community structure and rational use of mycorrhizal effect in Songnen saline-alkali grassland by human intervention in the future.

## 2. Materials and Methods

### 2.1. Study Site Description and Sample Collection

The study site is located in the southwest of Songnen Plain, Heilongjiang Province, China (125°22′–126°22′ E, 45°10′–46°20′ N). It belongs to the middle temperate continental monsoon climate and has a hot and rainy summer, cold and dry winter, and large temperature difference; the highest temperature in summer is 30 °C, the lowest temperature in winter is −25 °C, and the annual average temperature is 2.8 °C; and it has an annual frost-free period of approximately 140 days, annual evaporation of 1073.3–1368.4 mm, and annual precipitation of 350–550 mm mainly concentrated in July–September. *S. glauca* of the Amaranthaceae family and *P. tenuiflora* and *L. chinensis* of the Poaceae family are the main plant communities of the saline grassland of Songnen. Among them, *S. glauca* is the early succession (I.), *P. tenuiflora* is the middle succession (II.), and *L. chinensis* is the late succession (III.), which constitutes the succession series of the Songnen saline grassland community.

In August 2022, the roots and root enclosures of Alkali Plant, Star Grass, and Lamb Grass were collected in the saline-alkali grassland of Songnen City, Zhaodong City, Heilongjiang Province. We selected three repeat plots (10 m × 10 m), each of which had a minimum aggregation length of 100 m. Each site adopts the “multi-point mixed sampling method” [28] and the “five-point sampling method” [29], randomly sampling in five different directions, east, west, north, south, and middle. After removing the dead leaves and 5 cm thick topsoil, we dug a pit 10–30 cm deep and collected 1.5 kg of plant root and rhizosphere soil. The collected plant roots and rhizosphere soil were placed separately in sterile bags for cryopreservation at 4 °C and returned to the laboratory within 24 h. Samples were stored at an −80 °C ultra-low temperature for total DNA extraction within 30 days, and a portion of rhizosphere soil of 2 mm was screened for soil chemical analysis.

### 2.2. Soil Physical and Chemical Properties and Enzyme Activities

The chemical properties of the soil were determined by the methods of Boshidan [30], the enzyme activities were determined by the methods of Guan Songyin [31], and the soil protein associated with saccomycin was determined with the methods of Wright and David [32]. Soil pH value was measured by soil:water (1:5) with the PHS-3C instrument, EC value was measured by soil:water (1:4) with a DDS-11A conductivity meter, and soil organic carbon and organic matter were measured by potassium dichromate volumetric generation. Carbonate and bicarbonate were measured by phenolphthalein and sulfuric acid neutralization titration, soil total N was measured by the semi-micro kelvin method, total phosphorus and total potassium were determined by the NaOH melt-molybdenum antimony anti-colorimetric method, and total sodium, total calcium, and total magnesium in soil were determined by flame photometry. Sucrase activity was determined by 3,5-dinitrophenol colorimetry. We dissolved glucose in benzoic acid and configured it into a standard glucose solution (mg·mL^−1^), diluted it into a solution of different concentrations, added 3,5-dinitrophenol color development, and drew a standard curve colorimetric at a wavelength of 508 nm on a spectrophotometer. Urease activity was determined by sodium phenol colorimetry. After the N standard solution (1000 mg·L^−1^) was diluted at different times and sodium phenol and sodium hypochlorite were added for color development, the standard curve was developed on the spectrophotometer at the wavelength of 578 nm. Catalase activity was determined by potassium permanganate titration and, finally, easily extractable soil protein associated with saccomycin was determined by 20 mM citrate (pH = 7.0), and the difficult-to-extract soil protein associated with saccomycin was determined by 50 mM citrate (pH = 8.0). Bovine serum protein was configured into a standard protein solution, different concentrations of standard protein solution were added to Coomassie Brilliant Blue Reagent, and two minutes later, the standard curve was drawn on a spectrophotometer at a wavelength of 595 nm.

### 2.3. DNA Extraction and PCR Amplification

According to the E.Z.N.A.^®^ soil DNA kit (Omega Bio-tek, Norcross, GA, USA), the instructions were followed for the total DNA extraction of microbial communities. DNA extraction quality was detected using 1% agarose gel electrophoresis, and DNA concentration and purity were determined using NanoDrop2000; PCR amplification of the 18S rRNA gene fragment using AMV4-5NF (5′-AAGCTCGTAGTTGAATTTCG-3′) and AMDGR (5′-CCCAACTATCCCTATTAATCAT-3′) was as follows: Predenaturation at 95 °C for 3 min, 27 cycles (denaturation at 95 °C for 30 s, annealing at 55 °C for 30 s, extension at 72 °C for 30 s), then stable extension at 72 °C for 10 min. Finally, it was stored at 4 °C (PCR instrument: ABI GeneAmp^®^ 9700). The PCR reaction system was as follows: 5× TransStart FastPfu buffer 4 μL, 2.5 mM dNTPs 2 μL, upstream primer (5 uM) 0.8 μL, downstream primer (5 uM) 0.8 μL, TransStart FastPfu DNA polymerase 0.4 μL, template DNA 10 ng, ddH_2_O supplement to 20 μL. Each sample was repeated three times.

### 2.4. Illumina Miseq Sequencing

PCR products from the same sample were mixed and recovered using 2% agarose gel, purified using the AxyPrep DNA Gel Extraction Kit (Axygen Biosciences, Union City, CA, USA), electrophoresis assayed by 2% agarose gel, and quantified with Quantus™ Fluorometer (Promega, Madison City, WI, USA). We used the NEXTflexTM Rapid DNA-Seq Kit (Bioo Scientific, Austin City, TX, USA) for library construction: (1) Connector link; (2) use of magnetic bead screening to remove the connector self-connecting fragment; (3) enrichment of library templates by PCR amplification; and (4) magnetic beads recovered PCR products to obtain the final library.

### 2.5. Spore Morphological Identification

AMF spores in plant rhizosphere soil were separated by wet sieve decantation-sucrose centrifugation, and the Petri dish containing the sieve was placed under a double-tube solid anatomical microscope and AMF spores with the same morphological characteristics were transferred one by one to a 1.5 mL centrifuge tube and classified and numbered. After spores were prepared, their size, color, spore wall hierarchy, and thickness were observed under a light microscope, and the reaction after staining Melzer’s reagent was recorded. Referring to the Mycorrhizal Identification Manual [33] and the species descriptions and pictures provided on the international website of the International Arbuscular Mycorrhizal Fungal Collection (INVAM), the morphological preliminary search and judgment of AMF types around plant root circumference were carried out, and the separation frequency, relative abundance, and important values were calculated.

Distribution frequency (F) = number of occurrences of a genus or species of AMF/number of soil samples × 100%

Relative abundance (RA) = number of spores of a genus or species of AM fungus/total number of AMF at the sampling point × 100%

Average of Importance Value (IV) = (F + RA)/2 × 100%.

### 2.6. Determination of Root Colonization of Plants in the Wild and Establishment of Colonization System

The roots collected in the wild were cleaned, the lateral roots were cut into 1 cm root segments, and then the root segments were submerged in FAA fixative solution (5 mL formalin, 5 mL glacial acetic acid, and 90 mL of 70% alcohol) and fixed for 12 h. Observing the colonization of AMF in the root system with reference to the research method of Philips et al. [34], the root observation was repeated three times for each sample, and a total of 90 root segments were observed in each sample, and then MYCOCALC was used to calculate the colonization rate (%), colonization intensity (%), vesicle abundance, and arbuscular abundance (%).

The saline soil of Songnen was used as the cultivation substrate to establish a potting test colonization system. The soil samples and plant roots collected from the rhizosphere of the community of *S. glauca*, *P. tenuiflora*, and *L. chinensis* were used as in situ soil agents, and the soil-soaking solution filtered by the 38μm filter membrane was used as a bacterial filtrate to exclude bacterial interference. The seeds were collected from the saline grassland of Songnen, soaked in clean water, disinfected by 0.1% HgCl_2_, and sown at a low temperature of 48 h. A total of 5 groups were set up, two groups of controls (in situ soil and peaty soil) and three groups of experimental groups (*S. glauca* fungus, *P. tenuiflora* fungus, and *L. chinensis* fungus), and each group was repeated 5 times, for a total of 25 pots of seedlings, and all the sown pots were placed in the plant light culture chamber for cultivation. After 60 days of incubation, the fresh weight, dry weight, and root colonization status of each treated *L. chinensis* were tested, and the fresh weight (Wf) and dry weight (Wd) of the plants were accurately weighed using an electronic balance according to the method of Liu Ping and Li Mingjun [35]. The plant root colonization rate (%) and colonization intensity (%) refer to the methods of Philips et al. [34].

### 2.7. Data Analysis and Processing

The original sequencing sequence was quality controlled using fastp [36] (https://github.com/Open Gene/fastp, version 0.20.0, 19 May 2023) software, and splicing using FLASH [37] (http://www.cbcb.umd.edu/software/flash, version 1.2.7, 19 May 2023) software: (1) Filter the bases below the mass value of the tail of reads, set a window of 50 bp, and if the average mass value in the window is lower than 20, cut off the back-end base from the window, filter the reads below 50 bp after quality control, and remove reads containing N bases. (2) According to the overlapping relationship between PE reads, the paired reads are merged into a sequence, and the minimum overlap length is 10 bp. (3) The maximum allowable mismatch ratio of the overlap region of the splicing sequence is 0.2, and the screening does not conform to the sequence. (4) The sample is distinguished according to the barcode and primers at both ends of the sequence, and the sequence direction is adjusted, the number of mismatches allowed by the barcode is 0, and the maximum number of primer mismatches is 2. Using UPARSE [38] software (http://drive5.com/uparse/, version 7.1, 20 May 2023), OTU clustering of sequences based on similarity of 97% [39,40] and elimination of chimeras. For each representative sequence, the Greengenes database and RDP classifier (http://rdp.cme.msu.edu/,version 2.2, 20 May 2023) are used to annotate the species taxonomy of each sequence.

A one-way analysis of variance (ANOVA) was conducted to analyze all results using the SPSS 25.0 statistical analysis package. All data were presented as the mean ± standard deviation (SD) of the three independent replications, and Duncan’s multiple range test was applied with the level of significance fixed at *p* < 0.05. Excel was used for all calculations, and the results were expressed as the mean ± standard error (SE). Dry weight and fresh weight results were presented in histograms using SigmaPlot 14.0.

## 3. Results

### 3.1. Spore Morphological Identification of AMF

A total of 62 species of AMF in 17 genera were isolated in three succession stages (Table 1). Among them, the number of fungi in the *L. chinensis* stage was the largest, with 43 species, and 34 and 33 species were isolated and identified in the root enclosure soil of the *P. tenuiflora* stage and the *S. glauca* stage, respectively. *Glomus* and *Acaulospora* contain the largest number of species with 20 and 14 species. *Ambispora*, *Paraglomus*, *Sclerocystis*, *Racocetra*, *Rhizophagus*, *Dominikia*, and *Archaeospora* each identified 1 species. After calculation, the separation frequency, relative abundance, and important values were obtained, respectively. Furthermore, 92.59%, 8.90%, and 50.75% of the dominant species sp1 (IV ≥ 50%) were obtained in the succession process of Songnen saline-alkali grassland, but the existing morphological information could not classify it into specific species. In addition, the six most common strains (30% < IV < 50%) were obtained from *G. reticulatum*, *G. clarum*, *Ac. laevis*, *Cl. walkeri*, *Cl. claroideum*, and *Rh. intraradices* during the succession of pine saline grasslands. There were 18 common strains (10% < IV < 30%) and 36 rare strains (0% < IV < 10%). The specific spore image is supplemented with Figure S1.

### 3.2. Analysis of Soil Physical and Chemical Properties

We found differences in soil physicochemical properties, enzyme activity, and GRSP at different plant succession stages (Table 2). All plot soils were alkaline (pH 9.55–10.43), with conductivity ranging from 0.79 to 2.54 sm^−1^. pH and EC values decreased significantly with successional sequences, and the pH and EC values in the *S. glauca* stage were 0.09 and 2.22 times higher than those in the *L. chinensis* stage, respectively. In contrast, the organic matter, organic carbon, whole N, and total extracted GRSP were the highest in the *L. chinensis* stage, followed by the *P. tenuiflora* and *S. glauca* stages. Similarly, bicarbonate and total Mg remain stable in successional phases. The pH, EC, carbonate, whole K, all Ca, all Na, and urease stages of *L. chinensis* were lower than those in the stage of star grass and the *S. glauca* stage, while the soil proteins associated with organic matter, organic carbon, all N, all P, C/N, catalase, sucrase, and E-GRSP were higher than those in the stage of *P. tenuiflora* and *S. glauca* stage, which also indicated that the degree of soil salinization in the late succession stage was low and the nutrient content was high.

### 3.3. Changes in the Amount and Richness of AMF OUT

**Figure 1:**
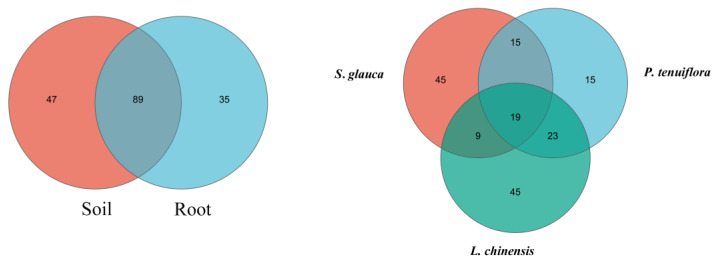
The status of identified OTU in rhizosphere soil, plant roots, and different successional stages.

**Figure 2:**
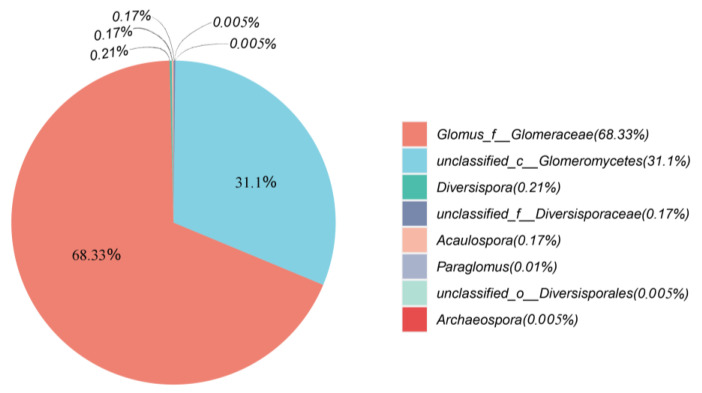
Distribution map of AMF in different genera based on OTUs.

Overall, a total of 387,703 Glomeromycota sequences were obtained from the samples using the AMV4.5NF/AMDGR primer pair, including 152,009 sequences from the *L. chinensis* community, 113,549 sequences from the *P. tenuiflora* community, and 122,145 sequences from the *S. glauca* community. Based on 97% species similarity, a total of 171 operational taxa (OTUs) were detected in the entire succession sequence community, of which the maximum number of OUT was 136 in the root enclosure and 124 in the plant root (Figure 1). In different succession stages, the maximum number of OUT in the *L. chinensis* community was 96, and the minimum OUT number in the *P. tenuiflora* community was 72. At present, the classification of the *Glomeromycota* is shown in Figure 2, and most AMF sequences belong to the *Glomus*, with 264,917, accounting for 68.33% of the total sequences obtained.

### 3.4. Changes in AMF Diversity and Community Structure of Plant Community Succession Sequence

**Figure 3:**
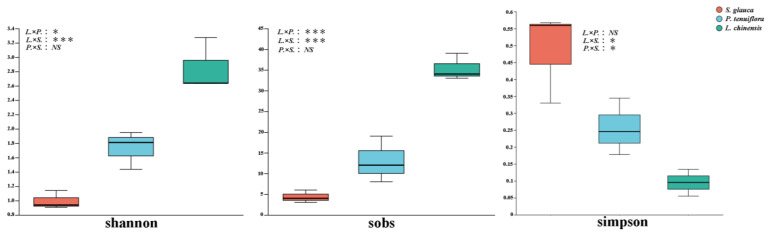
AMFα diversity in succession sequences of different plant communities. The green box represented *L. chinensis*, the orange yellow box represented *S. glauca*, and the blue box represented *P. tenuiflora*. *: *p* < 0.05; ***: *p* < 0.001; NS: *p* > 0.05. *L.*: *L. chinensis*; *S.*: *S. glauca*; *P.*: *P. tenuiflora*.

The dilution curve indicates that our sequencing depth is sufficient to cover most species in all samples (Supplementary Data Figure S2). The Shannon, Sobs, and Simpson indices were used to estimate the AMF α diversity between different succession stages (Figure 3). The results show that the Shannon index ranges from 0.93–3.27, the Simpson index ranges from 0.05–0.56, and the Sobs index ranges from 3–39. We found that the highest values of Shannon and Sobs diversity occurred in the *L. chinensis* stage, which was significantly higher than that in the other two stages (*p* < 0.05), and the lowest values of the Shannon and Sobs diversity indices occurred in the *S. glauca* stage. The maximum value of the Simpson index occurred in the *S. glauca* stage, which was significantly higher than that of the other two stages (*p* < 0.05), and the lowest value of the Simpson index occurred in the *L. chinensis* stage.

**Figure 4:**
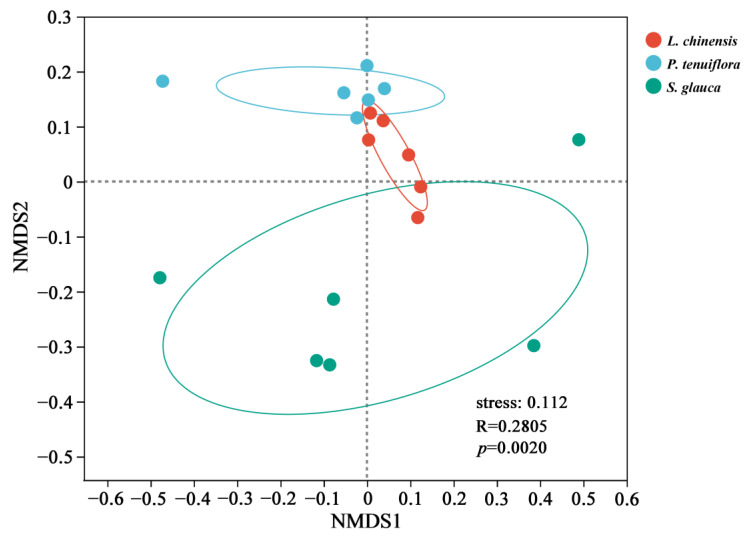
AMF community structure at different stages of plant succession. Red dot represented the *L. chinensis*, the green point represented the *S. glauca*, and the blue dot represented the *P. tenuiflora*. *p* < 0.05 was significant, *p* > 0.05 was not significant, and R was the variance.

Based on the UniFrac phylogenetic distance algorithm, all samples were clustered by NMDS analysis to detect the differences between the community structures of AMF at different succession stages. The results showed that there were significant differences in AMF community composition between different plant succession stages (Figure 4). There were obvious differences in AMF community structure between the *S. glauca* stage and the *P. tenuiflora* and *L. chinensis* stages, while the difference in AMF community structure in the *P. tenuiflora* and *L. chinensis* stages was not significant.

### 3.5. Effects of Environmental Factors on AMF Community Structure and Diversity

**Figure 5:**
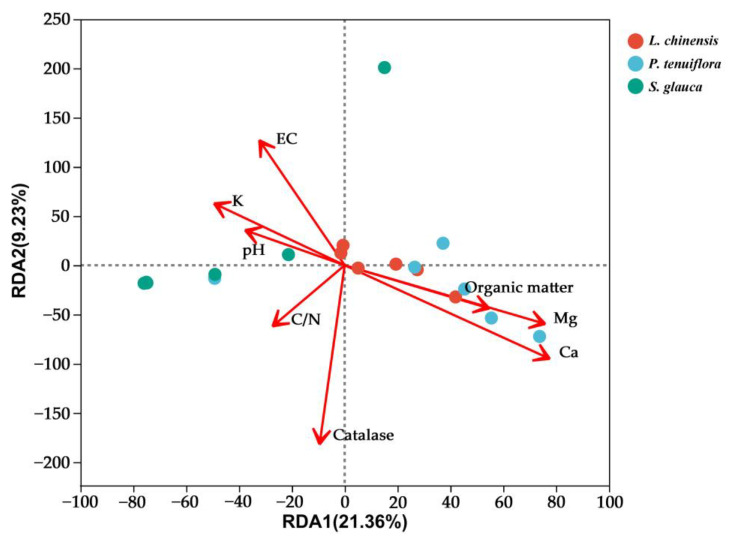
RDA analysis of soil physicochemical properties on AMF community structure. Red dot represented *L. chinensis*, the green point represented *S. glauca*, and the blue dot represented *P. tenuiflora*.

According to the results of the Mantel Test, some environmental factors significantly related to the AMF community structure were selected for RDA analysis (Figure 5). Analysis of VIF ANOVA showed that pH, EC, catalase, and Ca were important factors affecting the structure of AMF communities in all samples. The eigenvalues of the first and second axes are 21.36% and 9.23%, respectively, and the first two RDA axes explain 30.59% of the structural changes.

**Figure 6:**
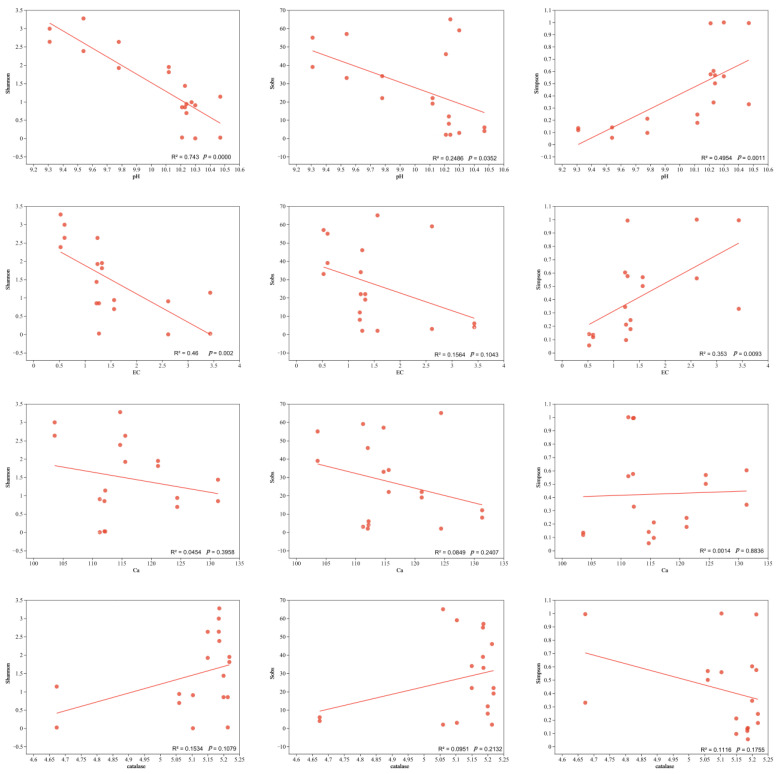
Linear regression analysis of soil physicochemical properties on AMFα diversity. *p* < 0.05 was significant, *p* > 0.05 was not significant, and R^2^ was the variance.

According to the results of RDA analysis, the four factors with the greatest influence on AMF community structure were obtained, and linear regression analysis was used for them and AMFα diversity (Figure 6). The results showed that pH had a significant correlation (*p* < 0.05) for Shannon, Sobs, and Simpson, and EC was significant for Shannon and Simpson (*p* < 0.05), but not for the Sobs index. Peroxidase and calcium ion content showed a certain correlation but not a significant effect on AMFα diversity.

### 3.6. Root Colonization of Plants in the Wild and Verifying the Ecological Matching Phenomenon

**Figure 7:**
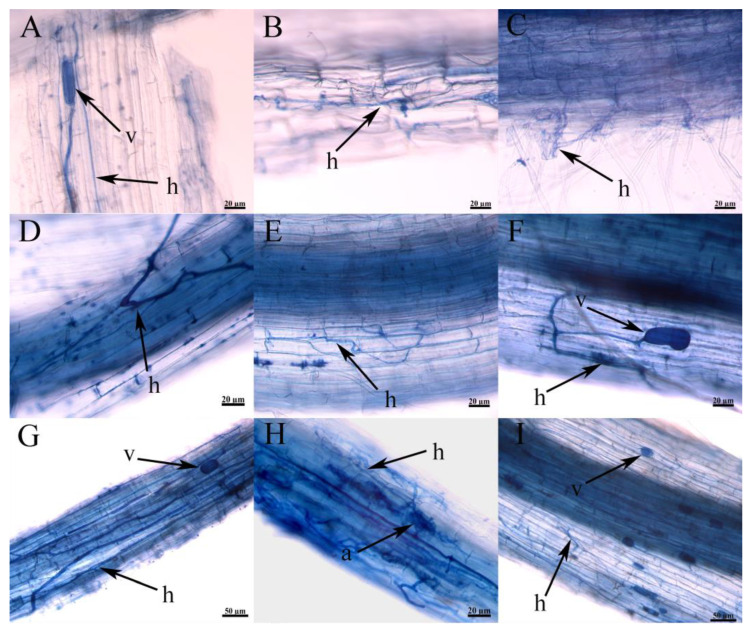
Colonization of AMF in the field on root systems of *S. glauca*, *P. tenuiflora*, *and L. chinensis*. (**A**–**C**) indicated the colonization structure of AMF in *S. glauca*. (**D**–**F**) indicated the colonization structure of AMF in *P. tenuiflora*. (**G**–**I**) indicated the colonization structure of AMF in *L. chinensis.* h—hyphae; v—vesicle; a—arbuscular.

Studies have shown that AMF successfully colonize the *S. glauca* community (Figure 7A–C), the *P. tenuiflora* community (Figure 7D–F), and the *L. chinensis* community (Figure 7G–I) that grow in the saline-alkali grassland of Songnen. AMF colonize the root from the outside of the host plant, and some hyphae expand into vesicles at the end, and some hyphae branch densely to form arbuscular. The mycorrhizal structure in the root segment of the *L. chinensis* community is rich, and the hyphae, vesicles, and arbuscular are densely distributed. Through the quantitative analysis of colonization of different plant samples (Table 3), the change trend of the AM fungal colonization rate was similar to that of soil salinity, and the difference in the AMF colonization rate between the three vegetation succession stages was significant (*p* < 0.05), and the succession of vegetation, the colonization rate (%), colonization intensity (%), vesicle abundance, and arbuscular abundance (%) increased gradually.

**Figure 8:**
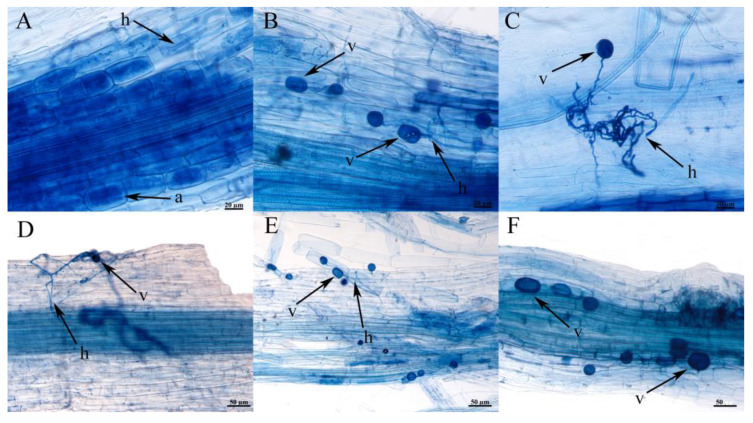
Colonization of AMF on root system of *L. chinensis* under different treatments in colonization system. (**A**,**B**) indicated the colonization structure of AMF in *L. chinensis* under YCJ treatment (saline-alkali soil was used as cultivated soil to inoculate AMF of *L. chinensis* stage). (**C**,**D**) indicated the colonization structure of AMF in *P. tenuiflora* under XXCJ treatment (saline-alkali soil was used as cultivated soil to inoculate AMF of *P. tenuiflora* stage). (**E**,**F**) indicated the colonization structure of AMF in *S. glauca* under JPJ treatment (saline-alkali soil was used as cultivated soil to inoculate AMF of *S. glauca* stage), h—hyphae; v—vesicle; a—arbuscular.

**Figure 9:**
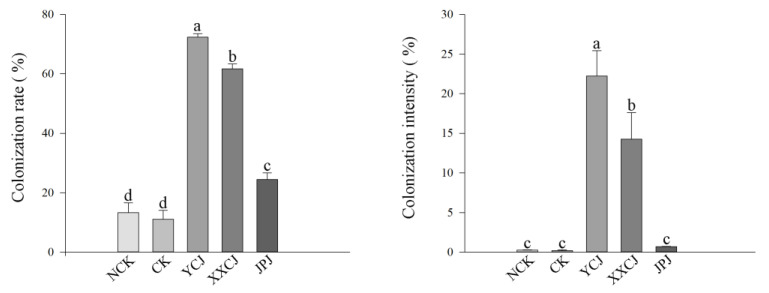
Effects of AMF on the colonization rate and colonization intensity of *L. chinensis* under different treatments. Values in the figure were mean ± standard error, and different lowercase letters indicated significant differences among different treatment groups (*p* < 0.05), NCK: Vegetative soil is used as cultivated soil and not inoculated with AMF, CK: Saline-alkali soil as cultivated soil and not inoculated with AMF, YCJ: Saline-alkali soil was used as cultivated soil to inoculate the AMF of *L. chinensis* stage, XXCJ: Saline-alkali soil was used as cultivated soil to inoculate AMF of *P. tenuiflora* stage, JPJ: Saline-alkali soil was used as cultivated soil to inoculate AMF of *S. glauca* stage.

**Figure 10:**
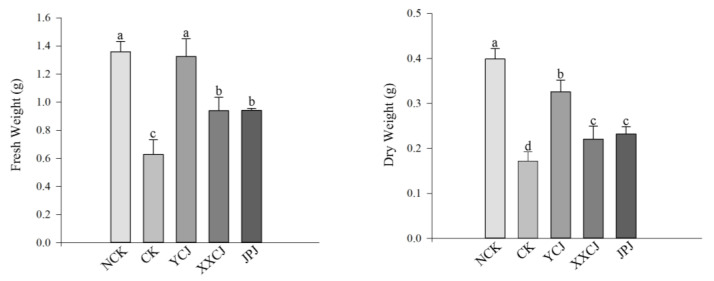
The dry weight and fresh weight of *L. chinensis* were changed under different treatments. Values in the figure were mean ± standard error, and different lowercase letters indicated significant differences among different treatment groups (*p* < 0.05), NCK: Vegetative soil was used as cultivated soil and not inoculated with AMF, CK: Saline-alkali soil as cultivated soil and not inoculated with AMF, YCJ: Saline-alkali soil was used as cultivated soil to inoculate AMF of *L. chinensis* stage, XXCJ: Saline-alkali soil was used as cultivated soil to inoculate AMF of *P. tenuiflora* stage, JPJ: Saline-alkali soil was used as cultivated soil to inoculate AMF of *S. glauca* stage.

After 60 days of the establishment of the colonization system of the late-successional plant *L. chinensis* with AMF communities of different successional stages, the colonization structures, such as hyphae, vesicles, and arbuscular, of AMF could be observed in plant roots (Figure 8A–F). Only a small amount of hyphae was observed in the two groups treated with bacterial filtrate during the same period, and there was no complete colonization structure. At the same time, it can be seen from Figure 9 that the colonization rate and colonization intensity of the late-successional AMF communities in the colonization test were the highest, followed by the middle and early successional AMF communities treatment, while the control group was the lowest, and the colonization rate and colonization intensity of the late-successional AMF communities treatment group were significantly higher than those of other treatments (*p* < 0.05). Similarly, as can be seen from Figure 10, both the dry weight and the fresh weight of the control group treated with peat soil were higher than those treated with in situ soil, and in in situ soil treatment, both the dry weight and the fresh weight of the post-succession AMF communities treatment group were significantly higher than those of other treatments (*p* < 0.05).

## 4. Discussion

Sixty-two species of AMF17 genera were isolated from the root enclosure soil of the three vegetation succession stages, and multiple species not reported in similar studies were found in this experiment, among which *Di. eburnea* and *Do. aurea* were isolated for the first time in the saline grassland of Songnen, indicating that there are abundant AMF resources in the saline habitat. *Glomus* identified the largest number of species, followed by *Acaulospora*, *Claroideoglomus*, and *Gigaspora* [41,42]. Among them, *Glomus* and *Acaulospora* were absolutely dominant in different succession stages, which was in line with the view of broad-spectrum symbiotic fungi [43]. Joinper and Abbott [44] found that different AMF had different tolerance to salt stress, and saline-alkali stress had little effect on *Glomeraceae* sp1. However, it had a certain degree of inhibition on the germination and bud tube elongation of dominant strains isolated from other plant root enclosures such as *G. mosseae*, *Cl. etunicatum*, and *Rh. intraradices* [45,46]. Since *Glomeraceae* sp1 image information had not been published in other habitat studies and its ecological function had not been explored, this strain had a strong survival ability in a saline-alkali environment, and the discussion of this strain should be strengthened in future studies.

Soil EC and pH are the main factors controlling the evolution of saline-alkali ecosystems [47]. As the first stage of the positive succession of the alkaline spots, the *S. glauca* community had the highest EC value, pH value, and carbonate and bicarbonate content, which also confirmed that the salinization degree of the *S. glauca* community was the highest. Total phosphorus content was lower in the three succession stages, which may be related to the presence of more carbonates and bicarbonates in the soil [48], and the presence of a large number of free carbonates and bicarbonates ions led to the conversion of soluble phosphorus into refractory phosphate compounds [30]. The contents of organic matter, organic carbon, and total N increased gradually with succession, and relevant studies showed that there were significant differences in the carbon and nitrogen of grassland in different regions and types [49]. These indexes were closely related to AMF activities, which might affect the secretion and release of some substances, and then affect the differences in the contents of organic matter, organic carbon, and total N at different succession stages. In addition, changes in organic matter, organic carbon, and total N content were closely related to the activities of urease, sucrase, and catalase. Urease could improve the strength of soil supply N [50], sucrase participated in improving the biotimeliness of organic matter and organic carbon [51], and catalase as an indicator of soil oxidation deg”ee s’owed a close correlation with soil organic matter and microorganisms [52]. Urease showed higher activity in response to the absence of the N element in the fields of *S. glauca* and *P. tenuiflora*, thus ensuring the supply of the N element. The activity of sucrase showed a trend of change according to the succession series, which may be related to the degree of salinization.

GRSP was a kind of soil glycoprotein produced during the decay of AMF mycelium, spores, and other propagators and was also a key component of soil organic matter and organic carbon in Songnen saline-alkali grassland [53]. In the later stage of succession, *L. chinensis* had a more developed root system than *S. glauca* and *P. tenuiflora* and could form a good co-effect with AMF so that AMF could absorb and use 20% of plant photosynthetic fixed carbon and transmit it to the soil through hyphae, spores, mycorrhizal residues, etc., and finally fix it in the soil in the form of balloonmycin-related soil protein to increase the carbon source in the soil [54]. The abundant rhizomycorrhizal network and the high content of soil protein related to saccomycin in the *L. chinensis* stage could adsorb or bind fine soil particles through physical action, form stable soil aggregates, improve the soil structure [55], and make the physical and chemical properties of soil in the late stage higher than those in the other two stages.

In this study, the abundance of AMF in soil and plant roots in Songnen saline grassland was unexpectedly higher than that of other ecosystems, such as agricultural ecosystems and forest ecosystems [56,57], indicating that Songnen saline grassland was rich in AMF compared with other ecosystems. Previous studies have also demonstrated that Glomus is widely distributed in different ecosystems, including grasslands and coastal wetlands [58,59]. The data of this study showed the same results, and the AMF community at different succession stages was dominated by the *Glomus*. It may be that the spores of the genus *Glomus* are relatively small, able to produce a large number of spores in a short period of time, and are easily transmissible [60], which is the same as ecological identification. The results of microbiota analysis showed that the Alpha diversity index and community structure composition of the *L. chinensis* stage were significantly different from those of the previous two stages, which may be due to the influence of soil environmental conditions, the salinization degree of the *L. chinensis* stage being the lowest, and the fact that it had a more suitable environment for AMF than the *S. glauca* and *P. tenuiflora* stages.

The EC value and pH showed a significant negative correlation with AMF diversity, which directly verified the claim that the salinization degree affects AMF growth and population distribution [61]. With the change in EC value and pH gradient, the α diversity and community structure of AMF changed significantly in different succession stages of Songnen saline grassland. On the one hand, it is because the higher EC can delay AMF spore germination and inhibit the elongation of AMF hyphae and the colonization of plant roots [62]. On the other hand, higher pH in soil affects the composition of plant species at different succession stages, which in turn affects AMF community structure [63]. In addition, catalase showed a positive correlation with AMF diversity, possibly because the samples with high enzyme activity removed harmful substances from the soil over time, avoiding damage to plant roots and AMF.

It is widely believed that there is a mycorrhizal symbiotic match (ecological fit) between the host plant and AMF [21,64]. Previous studies have shown that mycorrhizal function is dependent on plant origin, AMF source, and soil environment, and host plant biomass increases greatly when plants, AMF, and the soil environment are combined in the same domain [20,22]. In this study, when *L. chinensis* was inoculated with fungi at different succession stages, it was found that *L. chinensis* had a more significant effect on the biomass increase in host plants than other agents. This may be because *L. chinensis* stage fungi and *L. chinensis* host plants can form a better mycorrhizal symbiosis network system, expand the host plant root system, increase the effective use of N, P, and other elements in the soil by plant roots [65], and then make the *L. chinensis* stage fungal agent increase the biomass of host plants more significantly. The colonization rate and intensity of *L. chinensis* on host plants were higher than those in other treatment groups, which further indicated that AMF plants in Songnen saline-alkali grassland had mycorrhizal symbiosis matching phenomena (ecological adaptability).

## 5. Conclusions

In summary, the diversity and community structure of AMF at different succession stages in the Saline Songnen grassland were fully understood by morphology and high-throughput sequencing. The results showed that Glomus was the dominant genus of AMF in Songnen salin-alkali grassland. RDA analysis showed that the soil EC value, pH, calcium content, and hydroperoxide activity had the greatest influence on AMF community structure, and linear regression analysis showed that the EC value and pH had the greatest influence on AMF species diversity. In addition, in order to verify whether there is a mycorrhizal symbiotic match between AMF and host plants in the Songnen saline grassland, a colonization system was established. The results showed that the promotion effect of *L. chinensis* was more significant, indicating that there was a phenomenon of mycorrhizal symbiosis matching in Songnen saline-alkali grassland. Songnen saline grassland is an unique ecosystem, affected by a variety of factors, which provides a theoretical basis and data support to better understand the impact of environmental change on the structure and function of AMF in the Songnen saline-alkali grassland ecosystem.

## Figures and Tables

**Table 1:** Distribution of AMF strains in different succession stages.

Genus	Species	Stage			F%	RA%	IV%
		III	II	I			
*Glomus*	*G. multiforum*	+	+	+	37.04%	2.97%	20.01%
	*G. monosporum*	+			14.81%	0.82%	7.82%
	*G. badium*	+		+	25.93%	1.65%	13.79%
	*G. convolutum*	+		+	25.93%	2.14%	14.04%
	*G. reticulatum*	+	+	+	59.26%	4.61%	31.94%
	*G. microaggregatum*	+	+	+	18.52%	0.82%	9.67%
	*G. pansihalos*	+			7.41%	0.49%	3.95%
	*G. geosporum*	+		+	25.93%	1.48%	13.71%
	*G. halonatum*	+			7.41%	0.33%	3.87%
	*G. macrocarpum*	+	+		7.41%	0.33%	3.87%
	*G. hyderabadensis*	+			14.81%	0.66%	7.74%
	*G. reticulatum*	+	+	+	18.52%	0.99%	9.76%
	*G. lamellosum*	+	+		18.52%	0.82%	9.67%
	*G. clarum*	+	+	+	66.67%	6.10%	36.39%
	*G. fasciculatum*		+	+	29.63%	1.81%	15.72%
	*G. melanosporum*		+	+	22.22%	1.48%	11.85%
	*G. magnicaule*			+	3.70%	0.16%	1.93%
	*G. mosseae*		+		7.41%	0.33%	3.87%
	*G. fragile*		+		3.70%	0.16%	1.93%
	*G. versiforme*		+		3.70%	0.16%	1.93%
*Acaulospora*	*Ac. delicata*	+			22.22%	1.81%	12.02%
	*Ac. laevis*	+	+	+	62.96%	6.26%	34.61%
	*Ac. koskei*	+			11.11%	0.49%	5.8%
	*Ac. morrowiae*	+			25.93%	1.32%	13.63%
	*Ac. excavata*	+			11.11%	0.49%	5.8%
	*Ac. bireticulata*	+	+	+	40.74%	2.80%	21.77%
	*Ac. denticulata*	+			3.70%	0.16%	1.93%
	*Ac. lacunosa*	+			3.70%	0.16%	1.93%
	*Ac. gerdemannii*		+	+	7.41%	0.33%	3.87%
	*Ac. dilatata*			+	7.41%	0.33%	3.87%
	*Ac. paulinae*			+	3.70%	0.16%	1.93%
	*Ac. undulata*		+	+	7.41%	0.33%	3.87%
	*Ac. rugosa*			+	3.70%	0.16%	1.93%
	*Ac. mellea*		+		3.70%	0.16%	1.93%
*Claroideoglomus*	*Cl. walkeri*	+	+	+	70.37%	5.93%	38.15%
	*Cl. luteum*	+	+		40.74%	2.47%	21.61%
	*Cl. etunicatum*	+			7.41%	0.33%	3.87%
	*Cl. lamellosum*	+	+	+	51.85%	4.45%	28.15%
	*Cl. claroideum*	+	+	+	70.07%	6.75%	38.41%
*Gigaspora*	*Gi. decipiens*	+			7.41%	0.49%	3.95%
	*Gi. margarita*		+		3.70%	0.16%	1.93%
	*Gi. ramisporophora*		+		3.70%	0.16%	1.93%
*Septoglomus*	*Se. constrictum*	+	+	+	25.93%	1.65%	13.79%
	*Se. deserticola*	+	+		18.52%	0.99%	9.76%
*Funneliformis*	*Fu. mosseae*	+			3.70%	0.16%	1.93%
	*Fu. verruculosum*		+		3.70%	0.16%	1.93%
*Diversispora*	*Di. aurantia*	+			3.70%	0.33%	2.02%
	*Di. eburnea*	+	+	+	44.44%	2.80%	23.62%
*Pacispora*	*Pa. scintillans*	+	+	+	44.44%	2.97%	23.71%
	*Pa. chimonobambusae*	+			7.41%	0.33%	3.87%
*Scutellospora*	*Scu. reticulata*	+			3.70%	0.16%	1.93%
	*Scu. calospora*			+	3.70%	0.16%	1.93%
*Entrophospora*	*En. baltica*	+			7.41%	0.33%	3.87%
	*En. infrequens*			+	3.70%	0.16%	1.93%
*Archaeospora*	*Ar. leptoticha*		+	+	7.41%	0.33%	3.87%
*Dominikia*	*Do. aurea*		+	+	7.41%	0.33%	3.87%
*Rhizophagus*	*Rh. intraradices*	+	+	+	62.96%	5.11%	34.04%
*Racocetra*	*Ra. castanea*	+		+	25.93%	1.48%	13.71%
*Sclerocystis*	*Scl. sinuosa*	+		+	37.04%	3.46%	20.25%
*Paraglomus*	*Par. laccatum*	+	+		33.33%	2.31%	17.82%
*Ambispora*	*Am. leptoticha*	+	+	+	48.15%	3.46%	25.81%
	*Glomeraceae* sp1	+	+	+	92.59%	8.90%	50.75%

Note: *S. glauca* was the early succession (I.), *P. tenuiflora* was the middle succession (II.), and *L. chinensis* was the late succession (III.), +: at this stage there are stages, F: Distribution frequency, RA: Relative abundance, IV: Average of Importance Value.

**Table 2:** Soil physical and chemical properties, soil enzyme activity, and soil protein content related to saccomycin at each stage.

	*L. chinensis*	*P. tenuiflora*	*S. glauca*
pH	9.55 ± 0.21 c	10.19 ± 0.05 b	10.34 ± 0.10 a
EC (dS·m^−1^)	0.79 ± 0.34 c	1.55 ± 0.18 b	2.54 ± 0.81 a
OM (g·kg)	57.71 ± 5.09 a	21.59 ± 2.60 b	11.85 ± 6.74 c
OC (g·kg)	33.47 ± 2.96 a	12.52 ± 1.51 b	6.87 ± 3.91 c
CO_3_^2−^ (cmol·kg^−1^)	2.40 ± 1.41 b	6.98 ± 0.86 a	8.66 ± 2.68 a
HCO_3_^−^ (cmol·kg^−1^)	4.23 ± 3.15 a	3.28 ± 1.92 a	2.48 ± 1.72 a
N (g·kg)	1.43 ± 0.15 a	0.93 ± 0.17 b	0.39 ± 0.04 c
P (g·kg)	0.35 ± 0.09 a	0.30 ± 0.06 ab	0.28 ± 0.04 b
K (mg·kg)	21.85 ± 3.44 b	24.01 ± 0.81 ab	25.16 ± 1.72 a
Na (mg·kg)	144.28 ± 8.78 b	168.06 ± 5.92 a	165.61 ± 4.83 a
Ca (mg·kg)	111.31 ± 6.16 b	121.53 ± 8.53 a	115.97 ± 6.43 ab
Mg (mg·kg)	6.72 ± 0.52 a	6.60 ± 0.81 a	6.34 ± 0.65 a
C/N (g·kg)	23.50 ± 1.86 a	14.08 ± 4.12 b	17.52 ± 9.44 b
Catalase (μmol·d^−1^·g^−1^)	5.17 ± 0.02 a	5.21 ± 0.01 a	4.95 ± 0.21 b
Sucrase (mg·d^−1^·g^−1^)	0.33 ± 0.12 a	0.17 ± 0.02 b	0.10 ± 0.03 b
Urease (μg·d^−1^·g^−1^)	3.10 ± 0.96 b	4.51 ± 0.20 a	4.28 ± 0.17 a
E-GRSP (mg·kg)	0.43 ± 0.09 a	0.20 ± 0.06 b	0.15 ± 0.07 b
T-GRSP (mg·kg)	1.32 ± 0.16 a	0.65 ± 0.11 b	0.42 ± 0.08 c

Note: E-GRSP: Easily extractable soil protein associated with saccomycin, T-GRSP: Difficult to extract soil protein associated with saccomycin. Different letters in the line indicate that the index has significant differences between successive stages, with the maximum value marked as a, followed by b and c.

**Table 3:** The colonization of AMF in three succession stages.

	*L. chinensis*	*P. tenuiflora*	*S. glauca*
colonization rate (%)	92.592 ± 7.026 a	63.703 ± 11.110 b	33.333 ± 8.820 c
colonization intensity (%)	29.658 ± 11.727 a	5.030 ± 5.266 b	0.510 ± 0.425 b
arbuscular abundance (%)	2.954 ± 1.672 a	0.763 ± 1.760 b	0.008 ± 0.013 b
vesicle abundance (%)	8.774 ± 6.570 a	0.630 ± 1.639 b	0.038 ± 0.037 b

Note: Different letters in the line indicate that the index has significant differences between successive stages, with the maximum value marked as a, followed by b and c.

## Data Availability

Not applicable.

## Supplementary Materials

The following supporting information can be downloaded at: https://www.mdpi.com/article/10.3390/jof9090870/s1, Figure S1: Morphological identification of spores in soil. Figure S2: Dilution curve of species diversity.
