# Supplementary figures and images for: Electrical Conductivity and pH Are Two of the Main Factors Influencing the Composition of Arbuscular Mycorrhizal Fungal Communities in the Vegetation Succession Series of Songnen Saline-Alkali Grassland
Source: J Fungi (Basel). 2023 Aug 23;9(9):870. doi: 10.3390/jof9090870 (PMC10532779; doi:10.3390/jof9090870)

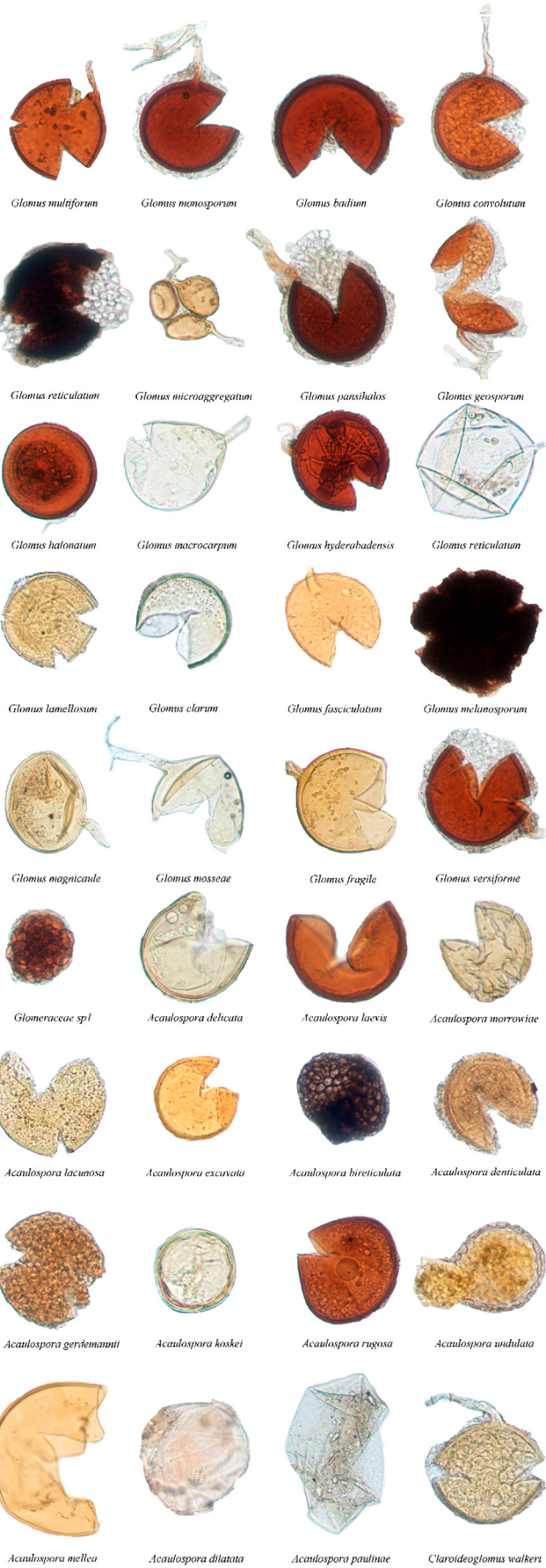

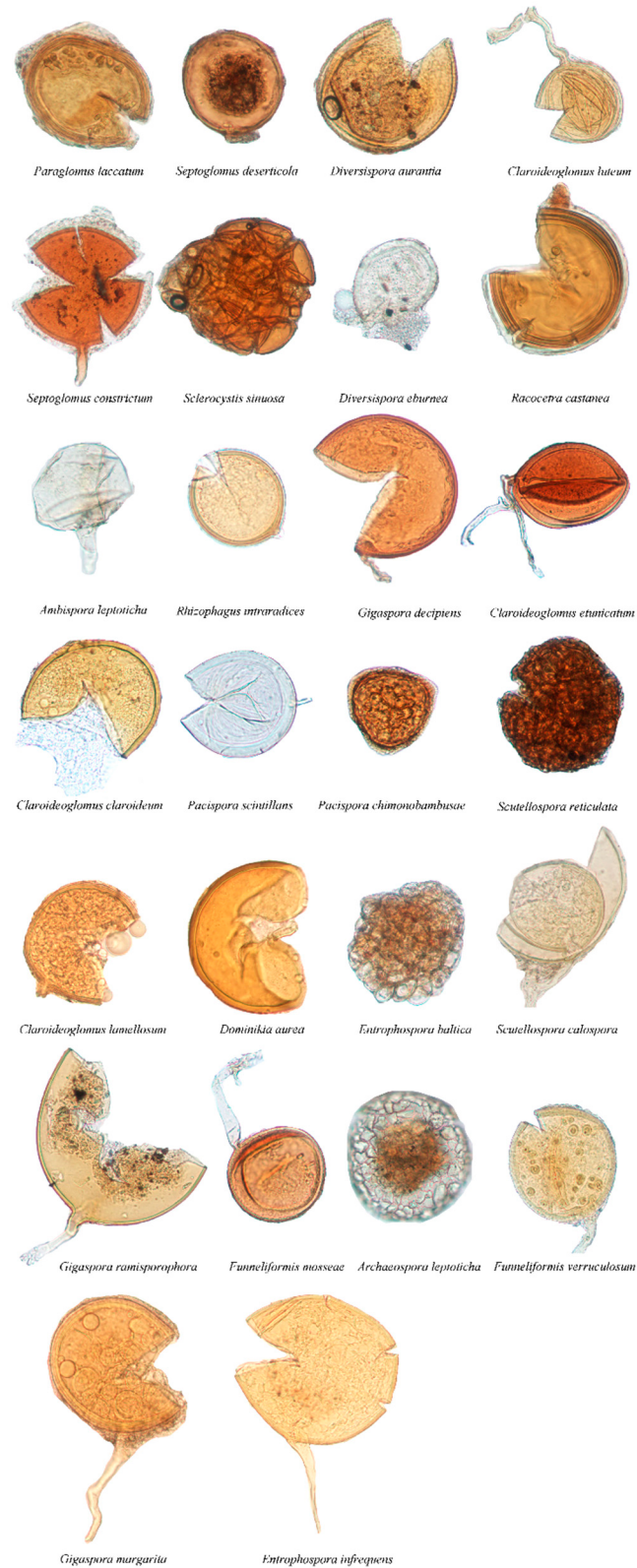

**Figure S1: Morphological identification of spores in soil**

Supplement: Supplementary file 1 [file jof-09-00870-s001.zip › Figure S1.pdf]

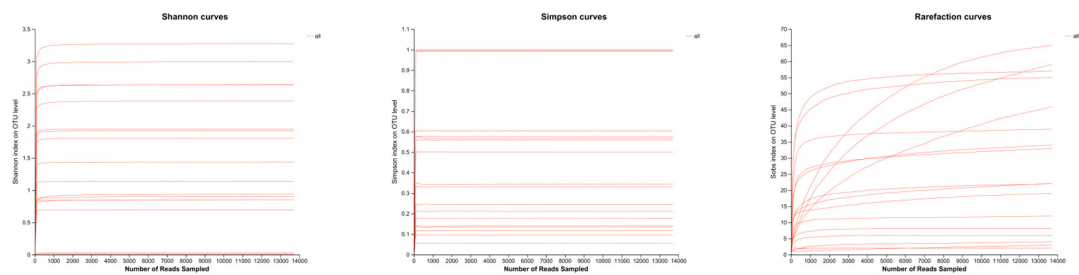

**Figure S2: Dilution curve of species diversity.**

Supplement: Supplementary file 1 [file jof-09-00870-s001.zip › Figure S2.pdf]
